# Case Report: A Rare Case of Coexisting of Autoimmune Polyglandular Syndrome Type 3 and Isolated Gonadotropin-Releasing Hormone Deficiency

**DOI:** 10.3389/fimmu.2021.734685

**Published:** 2021-09-14

**Authors:** Qiuhui Jiang, Ting Wu, Yuxian Zhang, Shunhua Wang, Liying Wang, Weijuan Su, Mingzhu Lin, Xuejun Li

**Affiliations:** ^1^The School of Clinical of Medicine, Fujian Medical University, Fuzhou, Fujian, China; ^2^Department of Endocrinology and Diabetes, Xiamen Diabetes Institute, Fujian Key Laboratory of Translational Research for Diabetes, The First Affiliated Hospital of Xiamen University, Xiamen, China

**Keywords:** autoimmune polyglandular syndrome type 3, secondary amenorrhea, isolated gonadotropin-releasing hormone deficiency, GnRH stimulation test, Graves’ disease, type 1 diabetes mellitus

## Abstract

APS (autoimmune polyglandular syndrome) is defined as the coexistence of at least two kinds of endocrine autoimmune diseases. APS type 3 comprises autoimmune thyroid diseases and other autoimmune diseases but does not involve autoimmune Addison’s disease. So far, APS-3 combined with isolated gonadotropin-releasing hormone (GnRH) reduction caused by the suspected autoimmune hypothalamic disease has not been reported. We recently received a 43-year-old woman with a one-year history of Graves’ disease (GD) and a four-month history of type 1 diabetes presented with hyperthyroidism and hyperglycemia. After the GnRH stimulation test, she was diagnosed with secondary amenorrhea attributed to suspected autoimmune Hypothalamitis and APS type 3 associated with Graves’ disease and Latent Autoimmune Diabetes (LADA). According to this case, the hypothalamus cannot be spared from the general autoimmune process. It is recommended to carry out the GnRH stimulation test when encountering APS patients combined with secondary amenorrhea.

## Introduction

APSs are rare conditions characterized by autoimmune activity against multiple endocrine organs, although non-endocrine organs can also be affected (1). To date, quite a few comorbidities have been described. Typical endocrine diseases include type 1 diabetes mellitus (T1DM), autoimmune thyroid disease, and Addison’s disease. Other frequently involved conditions comprise celiac disease, alopecia, vitiligo, hypogonadism, pernicious anemia, *etc.* APSs are generally categorized into four subtypes (2). APS type 1 is characterized by the development of at least two of three cardinal components comprising of chronic mucocutaneous candidiasis, hypoparathyroidism, and Addison’s disease; APS type 2 consists of Addison’s disease plus autoimmune thyroid disease or type 1 diabetes mellitus; APS type 3 is defined by the presence of autoimmune thyroid disease and another autoimmune illness but not Addison’s disease. Finally, APS type 4 refers to two or more organ-specific autoimmune disorders that did not fit into the characteristics of APS-1 through APS-3. We have recently received a patient with a rare combination of isolated GnRH deficiency suggesting autoimmune hypothalamic disease and APS type 3 complicated with Graves’ disease and LADA. To the best of our knowledge, this is the first case of such an association in humans. Herein, we present the clinical features and valuable diagnosis experience.

## Case Report

A 43-year-old woman with a one-year history of Graves’ disease (GD) and a four-month history of type 1 diabetes mellitus was admitted to our hospital with chief complaints of hyperthyroidism and hyperglycemia in March 2021. One year before admission, this patient was referred to the local hospital due to palpitation, emaciation, hyperhidrosis, ease of starving, *etc.* In addition to low thyroid-stimulating hormone (TSH), high free triiodothyronine (FT3), high free thyroxine (FT4), positive thyroid-stimulating hormone receptor antibody (TRAb), and a diffuse homogenous thyroid gland enlargement with increased blood flow by thyroid ultrasound was observed. Thus, GD was diagnosed. Then she started the treatment with antithyroid drugs (ATD). During the follow-up visits, the thyroid hormones were constantly high despite her treatment adherence. Five-month ago, she was treated with radioiodine therapy owning to the poor effect of ATD treatment. Since then, she had not taken antithyroid drugs. Three-month ago, the patient was hospitalized again in the local hospital and found elevated blood glucose due to the aggravation of hyperphagia, hunger and weight loss, *etc.* During hospitalization, she was subjected to a panel of laboratory examinations showing fasting insulin 8.6 uU/mL, fasting C-peptide 0.354 ng/mL, 2 hours postprandial insulin 15.2 uU/mL, 2 hours postprandial C-peptide 0.149 ng/mL, glycosylated hemoglobin (HbA1c) 7.9%, GAD antibody >2000 IU/mL. Accordingly, type 1 diabetes mellitus/LADA was diagnosed. She was started on premix insulin therapy twice daily, but her glucose control deteriorated, so she switched to basal/bolus insulin therapy.

The patient was 16 years old at menarche and had regular menstrual cycles. She had two children, both born in natural labor. She denied postpartum hemorrhage. In 2018, the disorder of the menstrual cycle began, along with a significant decrease in libido. In June 2020, the patient was menopausal (42 years old).

The patient had no history of autoimmune diseases such as vitiligo, autoimmune gastritis, pernicious anemia, neurodermatitis, alopecia areata, myasthenia gravis, systemic lupus erythematosus, autoimmune hepatitis, and rheumatoid arthritis. The patient’s mother, uncle, and grandmother had a history of type 2 diabetes. She had no family history of APS, autoimmune thyroid disease (AITD), or other immunological disorders.

Upon admission, her body mass index was 17.9 kg/m^2^, temperature 36.9°C, blood pressure 119/82mmHg, and pulse rate 98/min. On physical examination, she presented with a diffusely enlarged thyroid with moderate texture. No other obvious abnormality was observed. Results of laboratory tests were presented in **Table 1**. There was neither adrenal insufficiency nor hypocalcemia. Magnetic resonance imaging of her pituitary gland showed normal findings.

**Table 1:** Laboratory data on admission.

Variables	Results	Reference interval
Leukocyte count,10^9^/L	4.84	3.5-9.5
Erythrocyte count,10^12^/L	4.83	3.8-5.1
Haemoglobin,g/L	127	115-150
Platelet count,10^9^/L	157	125-350
Alanine aminotransferase (ALT), U/L	50.8	7-40
Aspartate aminotransferase (AST), U/L	44.7	13-35
Total Bilirubin,umol/L	21.2	0-21
Creatinine,umol/L	23	41-81
Urea,mmol/L	4.33	2.6-7.5
Sodium,mmol/L	137.5	137-147
Potassium,mmol/L	4.16	3.3-5.3
Calcium,mmol/L	2.31	2.11-2.52
Phosphorus,mmol/L	1.02	0.85-1.51
Parathyroid hormone (PTH), pg/mL	22.79	15.0-65.0
(25-OH) VitD3, ng/mL	28.67	>30
ALkaline Phosphatase (ALP), U/L	145	35-100
Glycated haemoglobin, %	14.6	3.8-6.5
C-P,ng/mL	0.435	1.1-4.4
GAD antibody	(+)	(-)
ICA antibody	(+ -)	(-)
Urine glucose	3+	(-)
Urine ketone	3+	(-)
Urine protein	1+	(-)
Insulin,pmol/L	96.01	16.5-84.7
HDL cholesterol,mmol/L	0.96	1.04-1.55
Triglycerides,mmol/L	0.75	0.4-1.82
Thyrotropin (TSH), mIU/L	0.005	0.55-4.78
Free triiodothytonine (FT3), pmol/L	12.96	3.5-6.5
Free thyroxine (FT4), pmol/L	41.02	11.5-22.7
Anti-thyrogloblin antibody (TgAb), IU/mL	466	0-40
Anti-thyroid peroxidase (TPO) antibody,IU/mL	>1000	0-35
TSH receptor antibody (TRAb), IU/L	>30	0-1.75
Cortisol,ug/dL	19.8	3.7-19.4
Adrenocorticotropic hormone,pg/mL	30.75	7.2-63.3
Progesterone (PRGE), ng/mL	0.78	0-0.73
estrogen (eE2), pg/mL	<11.80	0-32.2
luteinizing hormone (LH), mIU/mL	0. 42	15.9-54
Follicle stimulating hormone (FSH), mIU/mL	1.46	23-116.3
Prolactin (PRL), ng/mL	6.78	1.8-20.3
Testosterone (TSTII), ng/dL	30. 82	0-45.62

It is important to note that, in this case, E2 level decreased, but there were no increased FSH or LH levels following a one-year history of menopausal at the age of 42. It suggested that secondary ovarian dysfunction should be considered. Thus, we performed the magnetic resonance imaging of her pituitary gland, which showed normal findings, and a GnRH stimulation test [with triptorelin (Ferring Pharmaceuticals, Germany), 0.1 mg intravenously]. The results of the GnRH stimulation test were shown in **Table 2**. The GnH (FSH and LH) response to GnRH supported the diagnosis of isolated gonadotropin-releasing hormone deficiency. HLA Class II genotyping revealed DQB1*0201 allele and DRB1*0301-0803 gene site, and the corresponding genotypes were DQ2 and DR8. Based on the above clinical course and data, the patient was diagnosed with isolated GnRH deficiency and APS type 3 associated with Graves’ disease and LADA. Therefore, a hitherto unreported associated APS type 3 with isolated GnRH deficiency was described in this case report.

**Table 2:** The results of GnRH (Triptorelin, 100mg) stimulation test.

Time(min)	0 min	25 min	45 min	60 min	90 min	180 min
LH (mIU/mL)	0.57	2.91	3.18	3.51	3.63	3.54
FSH (mIU/mL)	1.57	8.12	9.27	10.1	13.6	15.37

During hospitalization, the patient chose to continue the ATD treatment (oral methimazole) rather than radioiodine therapy to control her thyroid disfunction. Doses were adjusted according to the levels of her thyroid hormones, and her thyroid function levels were relatively stable. As for the treatment of type 1 diabetes, we gave a subcutaneous insulin injection to control her blood glucose. Three days before admission, her fasting blood glucose fluctuated between 9 mmol/L and 11 mmol/L, and random blood glucose varied from 15 mmol/L to 20 mmol/L. After intensive insulin pump therapy for two weeks, her blood sugar was controlled within a range from 7 mmol/L to 13 mmol/L. Afterward, the insulin pump was replaced by glargine (subcutaneous injection) once daily combined with NovoRapid (subcutaneous injection) before meals. Doses were adjusted according to glucose monitoring level, clinical and biochemical response. During the outpatient follow-up, her random blood glucose varied from 7 mmol/L to 15 mmol/L. For premature menopause, previous studies (3, 4) have found that the lack of estrogen could lead to premature aging of blood vessels, bones, other tissues, *etc.*, and shorten the life expectancy of those patients. Therefore, estrogen replacement therapy (HRT) is recommended to reduce the adverse effects of low estrogen levels. Although we have repeatedly emphasized the importance of HRT treatment, the patient refused to receive HRT treatment because she was afraid of side effects.

## Discussion

**Figure 1:**
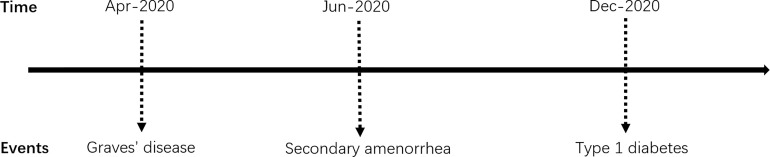
Timeline of the main clinical events.

APS type 3 is an adult type of APS, defined as the combination of AITD with other autoimmune diseases, except for Addison’s disease and hypoparathyroidism. Our patient’s autoimmune history began in 2020 at the age of 42 when she had been diagnosed with TRAb positive GD. Shortly afterward, it was followed by LADA. Thus the diagnosis of APS type 3 was established. Previous studies (5) have confirmed the frequent coexistence of T1DM and AITD in patients with APS. It should be highlighted that, in this case, the characteristic of APS type 3 was not typical initially because this patient manifested with hyperthyroidism rather than hypothyroidism (6). Therefore, it is not common for this patient to be diagnosed with GD. Furthermore, she did not improve after regular or even strengthened methimazole therapy. Even after radioactive iodine-131 therapy, deterioration of thyroxin levels control and weight loss progressed. On the other hand, there is normally a time gap for many years between the diagnosis of the first and second diseases among APS patients (5, 7). It turned out that the time gap between T1DM and AITD is the most extended (5). In terms of the sequence of endocrine gland insufficiency manifested in the APS type 3, it has been reported (8) that in 60% of APS type 3 patients, GD is more likely to occur before T1DM, with an average time of 7 years. In the cohort of autoimmune disease components in APS patients established by Martin P Hansen et al. (9), the onset of T1DM was earlier (average 27.5 years), while other component diseases appeared later, ranging from 36.5 to 40.5 years old. Other studies (10) have shown that the average onset age of T1DM in patients with Graves’ disease is 34 years old, with an incidence rate of 0.78%. However, in this reported case, GD and T1DM appeared almost simultaneously and developed rapidly, with the onset age relatively late (The time schedule of the events presents in **Figure 1**). Thus, the sequence of the affected autoimmune gland was unusual.

We should also consider that the patient’s current diagnosis as APS type 3 could be temporary. Patients with APS type 3 may eventually develop Addison’s disease and be reclassified as APS type 2. As related autoantibodies are usually detected a few years before the onset of the disease, we would like to put considerable emphasis on the necessity of the screen of the serology and function of other related autoimmune diseases linked. Due to the limited conditions, we did not measure the adrenal-associated 21-hydroxylase antibodies in this patient. Although the patient’s laboratory tests did not suggest clinical or biochemical abnormalities of adrenal dysfunction, it is still necessary to carefully monitor the function of the adrenal axis and screen the level of cortisol and electrolytes regularly in the future. Moreover, the association of endocrine autoimmune diseases is mainly attributed to common genetic susceptibility. APS type 3 is often observed in individuals in the same family. A study of 10 families with APS found that one in seven relatives had an undiagnosed autoimmune disease (11). Accordingly, to find hidden patients with endocrine diseases, their relatives should undergo screening for autoantibodies related to the disease components of APS, such as GAD antibody, IA-2A antibody, TPO antibody, 21-hydroxylase autoantibody, *etc.*

In consideration of whether the patient had other endocrine glands been affected, we noted that the patient’s amenorrhea age (42 years old) is earlier than the average. What makes it more intriguing was that the patient’s circulating blood FSH and LH levels were significantly lower than those women in physiological amenorrhea states. Thus secondary hypogonadism was considered. To confirm whether the decrease in GnH level was due to the pituitary dysfunction or secondary dysfunction of the hypothalamus, we conducted the magnetic resonance imaging of her pituitary gland, which showed normal findings. Moreover, the GnRH stimulation test had been carried out, showing that FSH and LH could be evidently stimulated, which indicated that secondary hypogonadism was caused by abnormal hypothalamic secretion of GnRH. However, when we evaluated the secretion function of other hormones in the pituitary gland (such as ACTH, PRL, *etc.*), there was no obvious abnormality, implying that the hypothalamic insufficiency was only manifested by isolated GnRH decrease. Taking into account all these factors, we may safely arrive at the diagnosis of isolated GnRH dysfunction. Heretofore, secondary hypogonadism has been reported in some APS patients, but most of them were considered to be caused by autoimmune hypophysitis (12–14) or poor control of Graves’ disease (15). Clinical cases of amenorrhea resulted from hypothalamic dysfunction/GnRH deficiency are mostly triggered by gene mutation, tumor, infiltration, trauma, *etc.* (16). Clinically, it is difficult to identify whether secondary hypogonadism is attributed to hypothalamic or pituitary diseases because it is impossible to directly detect the hormones secreted by the hypothalamus. Thus, apart from magnetic resonance imaging of the pituitary gland, we used the GnRH stimulation test to observe the response of the pituitary to GnRH and to determine the location of the lesion. Barkan et al. (12) had reported two patients with isolated gonadotropin deficiency after puberty. After completing the repeated GnRH stimulation experiments, blunted or absent responses of LH were seen, and the plasma level of FSH was undetectable. Finally, they reckoned that the decreased gonadal hormone resulted from autoimmune hypophysitis. Yet our patient’s GnRH stimulation test showed that FSH and LH could increase by more than six folds compared to baseline, especially FSH, which strongly implies that the pituitary responded well to GnRH. Moreover, the magnetic resonance imaging of her pituitary gland found no lesions such as a tumor, pituitary stalk deviation, inflammation, or infiltration. Thus, the lesion could be located in the hypothalamus, and it was rational to suspect that the isolated GnRH deficiency originated from the hypothalamus. The cause of the isolated GnRH dysfunction in our case was likely attributed to autoimmune. Given the property of multiorgan autoimmunity of APS type 3, it is not surprising that patients with these diseases are prone to the involvement of hypothalamus. In this circumstance, this is the first reported case of autoimmune isolated GnRH dysfunction associated with APS type 3.

It is well known that APS type 3 is a type of HLA-associated disease (17, 18). Many studies have indicated that HLA haplotypes DR3-DQB1*0201 and DR4-DQ*0302 contributed to the polyglandular autoimmune syndrome type 3 (17). The HLA profile of our patient manifested DQB1*0201 allele and DRB1*0301 and *0803 gene site, and the corresponding genotypes were DQ2 and DR8. DQ2 has been widely reported to correlate with APS (17). As for the HLA-DR8 haplotype, previous studies (19–21) have reported the potential associations between primary biliary cirrhosis, uneven NANB (non-A-non-B) hepatitis, and liver transplantation results. It is also significantly linked with Korean Graves’s patients with thyrotropin binding inhibiting immunoglobulins (TBII) and possibly related to the susceptibility gene that produces TSHR blocking antibodies (22). To the best of our knowledge, limited data are available on its association with the APS. The relations between HLA-DR8 and the components of the diseases in this patient remain to be elucidated.

Here we describe a middle-aged woman presenting with Graves’ disease, LADA, and isolated gonadotropin-releasing hormone (GnRH) deficiency. This is perhaps the first report of an APS type 3 patient complicated with isolated GnRH reduction possibly caused by the autoimmune lesions of the hypothalamus, which reminded us that the hypothalamus could not be spared from the general autoimmune process. Clinicians need to pay attention to the importance of carrying out the GnRH stimulation test when encountering APS patients manifesting secondary menopause and keeping an eye on the new onset of other autoimmune diseases.

## Data Availability Statement

The original contributions presented in the study are included in the article/supplementary material. Further inquiries can be directed to the corresponding author.

## Ethics Statement

Written informed consent was obtained from the individual(s) for the publication of any potentially identifiable images or data included in this article.

## Author Contributions

SW and WS provided clinical information, researched the data. YZ and LW performed the genetic analysis. QJ and TW wrote the draft of the manuscript. XL and ML provided critical discussion and reviewed/edited the manuscript. All authors contributed to the article and approved the submitted version.

## Conflict of Interest

The authors declare that the research was conducted in the absence of any commercial or financial relationships that could be construed as a potential conflict of interest.

## Publisher’s Note

All claims expressed in this article are solely those of the authors and do not necessarily represent those of their affiliated organizations, or those of the publisher, the editors and the reviewers. Any product that may be evaluated in this article, or claim that may be made by its manufacturer, is not guaranteed or endorsed by the publisher.
